# Chikungunya outbreak (2017) in Bangladesh: Clinical profile, economic impact and quality of life during the acute phase of the disease

**DOI:** 10.1371/journal.pntd.0006561

**Published:** 2018-06-06

**Authors:** Mohammad Sorowar Hossain, Md. Mahbub Hasan, Muhammad Sougatul Islam, Salequl Islam, Miliva Mozaffor, Md. Abdullah Saeed Khan, Nova Ahmed, Waheed Akhtar, Shahanaz Chowdhury, S. M. Yasir Arafat, Md. Abdul Khaleque, Zohora Jameela Khan, Tashmim Farhana Dipta, Shah Md. Zahurul Haque Asna, Md. Akram Hossain, KM Sultanul Aziz, Abdullah Al Mosabbir, Enayetur Raheem

**Affiliations:** 1 Biomedical Research Foundation, Dhaka, Bangladesh; 2 School of Environmental Science and Management, Independent University, Dhaka, Bangladesh; 3 Bangladesh University of Health Sciences, Dhaka, Bangladesh; 4 Department of Genetic Engineering and Biotechnology, University of Chittagong, Chittagong, Bangladesh; 5 Department of Microbiology, Jahangirnagar University, Dhaka, Bangladesh; 6 Uttara Women Medical College, Dhaka, Bangladesh; 7 Department of Medicine, Rajshahi Medical College Hospital, Rajshahi, Bangladesh; 8 National Institute of Cancer Research and Hospital, Dhaka, Bangladesh; 9 Department of Psychiatry, Bangabandhu Sheikh Mujib Medical University, Dhaka, Bangladesh; 10 Dhaka Medical College and Hospital, Dhaka, Bangladesh; 11 Bangladesh Institute of Research and Rehabilitation in Diabetes, Endocrine and Metabolic Disorders, Dhaka, Bangladesh; 12 Department of Microbiology, National Institute of Preventive & Social Medicine, Dhaka, Bangladesh; 13 Sir Salimullah Medical College Mitford Hospital, Dhaka, Bangladesh; 14 Department of Public Health Sciences, University of North Carolina at Charlotte, Charlotte, North Carolina, United States of America; Fundacao Oswaldo Cruz, BRAZIL

## Abstract

**Background:**

Chikungunya virus causes mosquito-transmitted infection that leads to extensive morbidity affecting substantial quality of life. Disease associated morbidity, quality of life, and financial loss are seldom reported in resources limited countries, such as Bangladesh. We reported the acute clinical profile, quality of life and consequent economic burden of the affected individuals in the recent chikungunya outbreak (May to September 2017) in Dhaka city, Bangladesh.

**Methods:**

We conducted a cross-sectional study during the peak of chikungunya outbreak (July 24 to August 5, 2017) to document the clinical profiles of confirmed cases (laboratory test positive) and probable cases diagnosed by medical practitioners. Data related to clinical symptoms, treatment cost, loss of productivity due to missing work days, and quality of life during their first two-weeks of symptom onset were collected via face to face interview using a structured questionnaire. World Health Organization endorsed questionnaire was used to assess the quality of life.

**Results:**

A total of 1,326 chikungunya cases were investigated. Multivariate analysis of major clinical variables showed no statistically significant differences between confirmed and probable cases. All the patients reported joint pain and fever. Other more frequently reported symptoms include headache, loss of appetite, rash, myalgia, and itching. Arthralgia was polyarticular in 56.3% of the patients. Notably, more than 70% patients reported joint pain as the first presenting symptom. About 83% of the patients reported low to very low overall quality of life. Nearly 30% of the patients lost more than 10 days of productivity due to severe arthropathy.

**Conclusions:**

This study represents one of the largest samples studied so far around the world describing the clinical profile of chikungunya infection. Our findings would contribute to establish an effective syndromic surveillance system for early detection and timely public health intervention of future chikungunya outbreaks in resource-limited settings like Bangladesh.

## Author summary

A major outbreak of chikungunya virus occurred for the first time in Dhaka, Bangladesh between May and September 2017. In this study, a face-to-face interview with a structured questionnaire was conducted to collect data to investigate the clinical symptoms, quality of life, and economic aspects of 1,326 chikungunya patients during the first two weeks of infection. The severity of the disease was similar to previously reported severe outbreaks elsewhere but joint pain prior to fever emerged as a unique symptom in the Dhaka outbreak. This unique clinical feature was consistent across age and sex of the patients. Some clinical symptoms varied with age. For instance, a higher proportion of skin rash were found among children (under 15) while morning stiffness, severity, and duration of pain were proportionally higher among other age groups. Joint swelling was most commonly noted in elderly patients (60+ years). About 83% of the patients reported low to very low overall quality of life (QoL) during first two weeks of chikungunya infection. Elderly patients reported lower average QoL scores compared to <60 years. Interestingly, housewives reported higher QoL score compared to those of businessmen and service holders. In particular, patients in the highest monthly income category bracket (BDT 50,000 per month; >$606 per month) reported the lowest average overall score. Nearly 95% of the patients have mostly confined to sickbed and approximately 30% of them lost more than 10 days of productivity due to severe arthropathy. Our study would contribute to establishing an effective syndromic surveillance system for early detection and timely public health intervention of future chikungunya outbreaks in resource-limited countries like Bangladesh.

## Introduction

Chikungunya is a mosquito-borne (*Aedes species)*, self-limiting, febrile illness with severe debilitating arthropathy caused by chikungunya virus (CHIKV). This virus was first identified during an epidemic of febrile polyarthralgia in Tanzania in 1953 [1]. Since then, CHIKV has been reported to cause several large-scale outbreaks in Africa, India, Southeast Asia, Western Pacific and Americas [2–4]. Before 2000, chikungunya outbreaks were mostly sporadic and limited. But thereafter, the virus has been frequently causing severe forms of epidemics imposing heavy economic burden and productivity loss [5,6]. The CHIKV outbreaks are characterized by a sudden disappearance for a considerably long period of time from a particular geographic area before re-emergence. As such, chikungunya has re-emerged in devastating form of epidemics in and around the Indian Ocean in 2005, nearly after 30 years of quiescence [7].

In Bangladesh, the first recognized outbreak of chikungunya was reported in 2008 in two villages in the northwest part of the country adjacent to Indian border [8]. Two small-scale outbreaks were documented in rural communities in 2011 [9] and 2012 [10]. Dhaka, the capital of Bangladesh, is one of the most densely populated cities in the world with approximately 18 million inhabitants [11], have experienced a large-scale chikungunya outbreak in 2017. Mainstream local media outlets have extensively covered the epidemic [12,13].

Despite a lower middle-income country, Bangladesh has achieved remarkable progress in reducing maternal and child mortality by improving primary healthcare access [14]. However, country’s overall public healthcare facilities are not adequate. Approximately 70% patients prefer private clinics/hospitals for seeking medical advice [15,16]. It allocates only US$31 per capita on health expenditure [17]. Thus, continuous surveillance of any unprecedented disease like chikungunya is a huge challenge in Bangladesh since most public hospitals lack modern diagnostic facilities and medical documentation system [18]. Therefore, data from public medical facilities alone cannot provide an overall picture of an outbreak resulting in underreporting of cases. A state-run institute named Institute of Epidemiology, Disease Control and Research (IEDCR) has monitored the recent chikungunya outbreak based on RT-PCR data sourced from three diagnostic laboratories including the institute itself and two other private facilities. According to IEDCR’s own data on 1003 RT-PCR confirmed cases, the peak of the outbreak was between early May and end of July 2017 [19]. Also, according to their newsletter, about 87.3% (12060 out of 13814 cases) of suspected chikungunya patients visited three public hospitals (DMCH, ShMCH, SSMCMH) in addition to IEDCR for treatment. Taking other public hospitals and more than 2000 registered private clinics/hospitals in the Dhaka metropolitan are into account [20,21], arguably, there was a widespread chikungunya outbreak in the city.

CHIKV infection has emerged as a major public health concern since it often affects a large proportion of the population within an outbreak area and causes considerable pain, distress, and anxiety as well as significant economic burden due to severe clinical manifestations [22–25]. CHIKV infection is usually non-fatal and the sign of clinical symptoms resolves over time. The clinical severity of this disease is associated with reduced quality of life (QoL). It is found that QoL drops dramatically when patients are severely affected, especially during the early phase of chikungunya infection lasting for one or two weeks, while prolonged musculoskeletal manifestations (chronic arthralgia) may last for months to years [23]. A study, for instance, has observed a 20-fold reduction of QoL in the non-recovered CHIKV positive group while 5-folds reduction was in recovered group as compared to healthy control group [26].

Poor QoL resulting from CHIKV is linked to immediate and long-term economic burden [24,27,28]. Due to lack of health insurance system and other socio-economic factors, it is challenging to determine the actual economic burden of chikungunya outbreaks in developing countries. It appears that the actual economic loss is often overlooked because of non-fatality of the disease. CHIKV outbreaks have caused significant financial burdens on society level in India and across Latin America [24,29–31]. The impact on household finance was substantial, particularly during acute phase of infection when QoL was found to be at the lowest level. The poor segment of the population generally bears the economic burden most. Consequently, it may aggravate poverty. Long-term arthralgia secondary to CHIKV has also reported to cause significant economic impact [24]. A higher health care utilization has been reported for patients up to six years after acute infection of CHIKV [27]. In this study, we are interested to find out the answers for two primary research questions: a) the impact of clinical severity on QoL during the acute phase, and b) the impact of treatment cost on the economic conditions of CHIKV patients during acute phase.

## Methods

### Case definition

In our study, we investigated patients who experienced typical clinical symptoms of CHIKV infection [32] (febrile illness with arthralgia/arthritis) during the peak of recent Dhaka-outbreak (May-July, 2017). Patients with a positive RT-PCR or serological test (CHIKV positive but not dengue) were categorized as confirmed cases. For probable case, we followed the recommendation of the World Health Organization (WHO) [33] and standardized European case definition [34] where during an established outbreak, a patient meeting both clinical and epidemiological criteria are considered as probable case. Thus, patients diagnosed by medical practitioners based on characteristic clinical features of CHIKV infections without a RT-PCR or serological tests were documented as probable cases. As discussed earlier, because of the magnitude of the outbreak, the local health authority (IEDCR) monitored the outbreak and released health bulletin on a daily basis. There was no sign or warning of any other arboviruses related outbreaks (such as dengue) by IEDCR. Unlike developed countries, our healthcare system lacks a functional referral system [18] and organized medical record-keeping system. Therefore, we reached out to every enrolled patient to verify the documents of CHIKV diagnosis. Scanned copies of the laboratory test results were collected from the confirmed cases as a proof. Patients claimed to have CHIKV infection without physicians’ confirmation (clinical and or laboratory) were excluded from the study. It is important to note that molecular and serological diagnostic tests for CHIKV infection were available only in a handful of diagnostic facilities (mostly private). The cost of diagnostic test was relatively high considering the average income of city dwellers. In this context, given the insignificant mortality related to CHIKV, the government health regulatory authority (DGHS, Bangladesh) discouraged (non-obligatory instruction) suspected patients for laboratory tests in order to turn down the panic among city dwellers [35]. As a result, we had more probable cases than confirmed cases.

### Study design and data collection

A cross-sectional study was conducted between July 24 and August 5, 2017, to investigate the clinical profiles, economic burden, and quality of life of chikungunya affected individuals. Biomedical Research Foundation (BRF), Bangladesh took the initiative to study the impact of this widespread outbreak. The study was conducted by a team of 111 volunteer researchers comprising of clinicians, public health professionals, statisticians as well as undergraduate and postgraduate students. Considering the urgency of the issue and time-sensitive nature of the outbreak, we relied entirely on voluntary services rather than financial support from external sources. Participants were conveniently selected by the members of the research team from within their known circles (e.g., friends, friends of friends) living in Dhaka city, where the outbreak has occurred. Data were collected via face-to-face interview using a structured questionnaire. Enumerators were given one-day training on the use of the questionnaire for face-to-face interviews. Respondents were asked about clinical symptoms, loss of productivity due to missing work hours, and quality of life during their first two-weeks of symptom onset. Data on economic variables were collected from only the earning members of the chikungunya affected families.

### Statistical analysis

Descriptive and inferential statistical procedures were used to ascertain the clinical profile of CHIKV infection and its impact on quality of life and economic well-being. Descriptive statistics were reported as percentage and means when applicable along with standard deviation. The intensity of joint pain was evaluated by a 10-point numerical rating scale (NRS), which was categorized as mild (score between 1 and 3), moderate (score between 4 and 6) and severe (score between 7 and 10) in the analytic stage [36]. To determine the impact of chikungunya infection on quality of life (QoL), World Health Organization (WHO) endorsed quality of life questionnaire (brief version), known as WHOQOL-BREF, was used. We used the validated Bengali version of the questionnaire [37]. The responses from WHOQOL-BREF questionnaire were analyzed as per the recommendation and scoring guidelines [38]. Cronbach's alpha coefficient was calculated to check the internal consistency of scores. For assessing the impact of CHIKV on the economic well-being of a family, a 10-point rating scale questions were used to capture granular responses. Missing cases were excluded from bivariate analysis.

Completed data collection forms were scrutinized by data collection supervisors. Follow up calls were made within two days of data collection to 10% randomly selected participants to ensure the authenticity of the responses. Verified data were entered and subsequently managed using REDCap electronic data capture tool hosted at BRF [39].

Data were analyzed using R statistical software. For testing association between categorical data, Pearson’s chi-square test was used, and Yate’s correction for continuity was applied where appropriate. We performed independent sample t-test when comparing means of continuous variables. A two-tailed p-value smaller than 0.05 was considered statistically significant.

### Ethical consideration

The study was approved by the Ethical Review Committee (ERC) of Bangladesh University of Health Sciences (Memo no: BUHS/BIO/EA/17/077). As approved by ERC, verbal and/or written informed consent was obtained from every participant as per their convenience. Trained enumerators first approached and explained consent form to the prospective participants and study questionnaire was shared or discussed with them. After obtaining consent (oral and/or written), participants were registered for face-to-face interview. Some respondents could not sign their names, in which case the questionnaire was marked indicating a case with verbal consent. All adult participants 18 years or above provided informed consent. Although children (18 years and below) were included in the survey, no child was interviewed. Parents or guardians provided information about children’s clinical symptoms. Children were also not included in the economic wellbeing and quality of life section of the study.

## Results

### Participants

**Fig 1:**
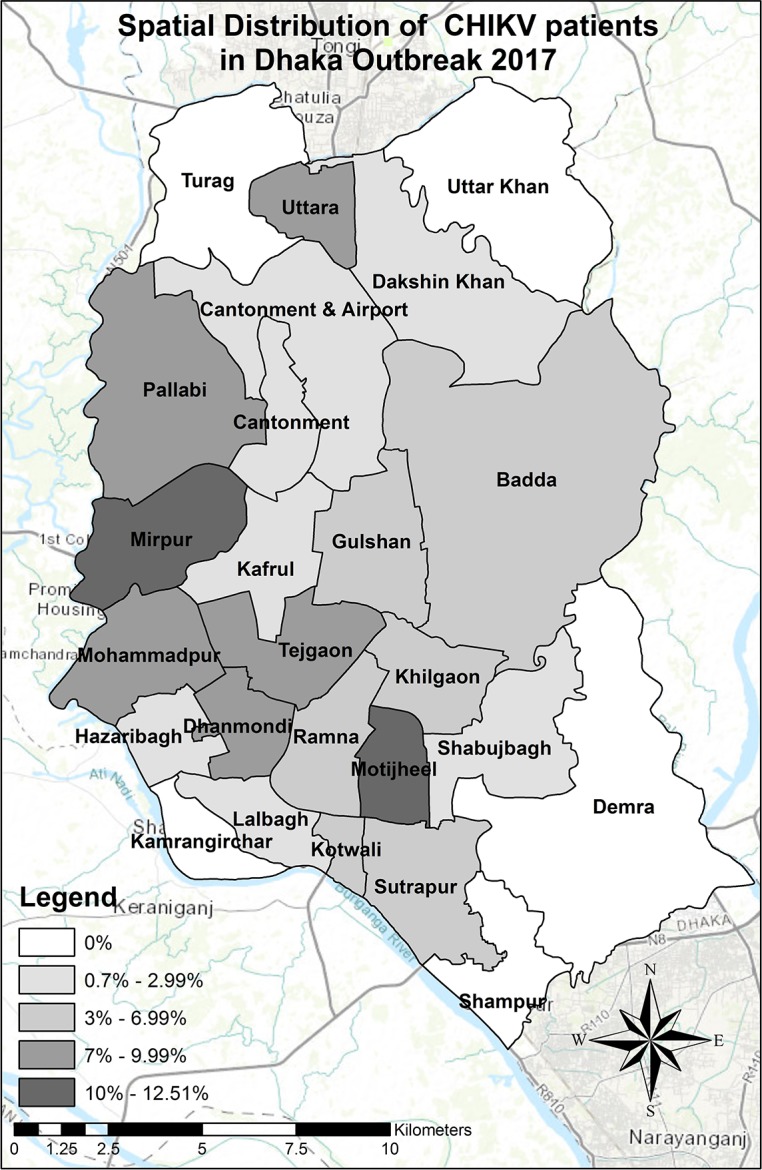
Spatial distribution of 855 patients out of 1326 CHIKV infected patients in Dhaka city. The remaining 471 patents were not interested to disclose their address during the interview. Data presented in this Figure as % of total patients had no reservation to share their residential address (n = 855).

A total of 1,474 patients were enrolled in our study. After rigorous verification and cross-checking, 148 cases (10%) were discarded due to the incompleteness of data. Finally, of all 1326 verified cases, 18% (239/1,326) constituted confirmed cases (214 cases were serologically confirmed, while 25 cases by RT-PCR) and 82% (1087/1326) were probable cases. The geospatial distribution of 855 patients (who provided address) out of 1326 showed that our study represented most of the administrative zones (20 out of 25) of Dhaka City (Fig 1). The mean age of the participants was 33.74 years (SD = 14.83) and male to female ratio was 1.33:1. Children (<15 years of age) represented 6.4% of the study subjects, 42.5% were adolescents and young adults (AYA, 15–29 years), 44.3% were adults (30–59 years) and 6.8% of the cases aged over 60 years (Table 1). About 43.7% of the participants were graduates, 33.3% completed high school and 4.9% had no education. A total of 339 (25.6%) participants had comorbidities (Table 1). Seventy-six patients (5.7%) were hospitalized with chikungunya infection, of which 35 were confirmed cases.

**Table 1:** Baseline demographic characteristics of patients (n = 1326).

Variable		Total cases	Confirmed cases	Probable cases	χ^2^	p
Total cases		1326	239 (18.02%)	1087 (81.98%)		
Age in year						
	Mean (SD)	33.74 (14.83)	37.18 (15.79)	32.98 (14.50)		
Age group (in year)						
	Children (<15)	85 (6.41%)	12 (5.02%)	73 (6.72%)	22.44	<0.0001
	AYA (15–29)	563(42.46%)	76 (31.8%)	487 (44.8%)		
	Adult (30–59)	588 (44.3%)	123 (51.5%)	465 (42.8%)		
	Elderly (>59)	90 (6.79%)	28 (11.72%)	62 (5.7%)		
Gender						
	Male	758(57.16%)	126 (52.72%)	632 (57.56%)	2.352	0.072
	Female	568(42.84%)	113 (47.28%)	455 (41.44%)		
Marital status						
	Married	785 (59.2%)	167 (69.87%)	618 (56.85%)	13.752	<0.0001
	Single	541 (40.8%)	72 (30.13%)	469 (43.15%)		
Education						
	No education	65 (4.9%)	5 (2.1%)	60 (5.5%)	39.308	<0.0001
	Primary	241 (18.2%)	32 (13.4%)	209 (19.2%)		
	Secondary	441 (33.3%)	61 (25.5%)	380 (35%)		
	Graduate	579 (43.7%)	141 (59%)	438 (40.3%)		
Employment status						
	Business	132 (9.95%)	15 (6.28%)	117 (10.76%)	48.01	<0.0001
	Housewife	214 (16.14%)	47 (19.67%)	167 (15.36%)		
	Retired	37 (2.79%)	16 (6.69%)	21 (1.93%)		
	Service	453 (34.16%)	94 (39.33%)	359 (33.03%)		
	Student	387 (29.19%)	58 (24.27%)	329 (30.27%)		
	Others	103 (7.77%)	9 (3.77%)	94 (8.65%)		
Income						
	<10,000 BDT (<$121)	323 (24.4%)	32 (13.4%)	291 (26.8%)	75.348	<0.0001
	10,000–24,999 BDT ($121-$303)	380 (28.7%)	46 (19.2%)	334 (30.7%)		
	25,000–49,999 BDT ($304-$606)	327 (24.7%)	60 (25.1%)	267 (24.6%)		
	> = 50,000 BDT (> = $606)	296 (22.3%)	101 (42.3%)	195 (17.9%)		
Co-morbidities						
	Total	339 (25.6%)	74 (31%)	265 (24.4%)	4.462	0.035
	Hypertension	167 (12.6%)	19 (7.9%)	148 (13.6%)	5.713	0.017
	Diabetes	125 (9.4%)	12 (5%)	113 (10.4%)	6.629	0.010
	Stroke	9 (0.7%)	0 (0%)	9 (0.8%)	1.992	0.158
	Heart Disease	40 (3%)	1 (0.4%)	39 (3.6%)	6.727	0.009
	CKD	8 (0.6%)	0 (0%)	8 (0.7%)	1.77	0.183
	COPD	9 (0.7%)	2 (0.8%)	7 (0.6%)	0.108	0.742
	Others	91 (6.9%)	16 (6.7%)	75 (6.9%)	0.013	0.91
Hospitalization		76 (5.7%)	35 (14.6%)	41 (3.8%)	42.87	0.000

### Clinical profile

Over 90% of the respondents reported high-grade fever with an average maximum temperature of 103.6°F (SD = 0.89). The mean duration of fever was 4.88 days (SD = 2.7). As the first clinical symptom, 74.6% (n = 1326) of the respondents experienced pain (joint and/or muscle pain) prior to fever (Table 2). This unique clinical feature was consistent irrespective of age and sex of the patients. About 85% of the patients (both confirmed and probable cases) complained severe pain with a mean pain score of 8.3 (out of 10) (SD = 1.63), and about 65% patients suffered more than 10 days during the acute phase (Table 3). Arthralgia was oligoarticular (2–4 joints) in 40.1%, and polyarticular (>5 joints) in 56.3% of the patients. Peripheral small joints were the most common site of involvement. The joint pain was symmetrical in 64.8% of the patients (Table 3). Joint swelling and skin rash were significantly higher among confirmed cases (62.6% and 78.2% respectively). Other common symptoms reported were redness of eyes (over 56.5%), nausea (60%), oral ulcer (31.2%), diarrhea (25%), and edema (18%) (S1 Table). Bivariate analysis showed that the severity of some clinical symptoms was gender (S1A Fig) and age group specific (S1B–S1E Fig). For other clinical symptoms analyzed for Dhaka outbreak, see S1 Table and S2 Table.

**Table 2:** Clinical profile of chikungunya patients (n = 1326) in Bangladesh.

Variable	Total cases	Confirmed cases	Probable cases	P
Arthralgia	1326 (100%)	239 (100%)	1087 (100%)	
Pain before fever	990 (74.66%)	177 (70.05%)	813 (74.79%)	
Skin Rash	923 (69.6%)	187 (78.2%)	736 (67.7%)	0.001
Itching	807 (60.9%)	148 (61.9%)	659 (60.6%)	0.709
Headache	1025 (77.3%)	165 (69%)	860 (79.1%)	0.001
Myalgia	919 (69.3%)	155 (64.9%)	764 (70.3%)	0.099
Edema	239 (18%)	53 (22.2%)	186 (17.1%)	0.065

**Table 3:** Arthralgia profile of chikungunya patients (n = 1129, cases with past history of arthritis were excluded) in Bangladesh.

Variable		Total cases	Confirmed cases	Probable cases	χ^2^	P
Joint pain		1129 (100%)	198 (100%)	931 (100%)		
Joint stiffness		838 (74.22%)	142 (71.71)	696 (74.76%)	0.789	0.374
Joint swelling		589 (52.17%)	124 (62.62%)	465 (49.95%)	10.65	0.001
Arthralgia						
	Oligoarthralgia	453 (40.1%)	89 (44.9%)	364 (39.1%)	2.36	0.307
	Polyarthralgia	636 (56.3%)	103 (52%)	533 (57.3%)		
Pain score (NRS)						
	Mild	9 (0.8%)	0 (0%)	9 (0.97%)	2.11	0.348
	Moderate	152 (13.46%)	25 (12.63%)	127 (13.64%)		
	Severe	968 (85.74%)	173 (87.37%)	795 (85.39%)		
Duration of pain in first two weeks						
	<7 days	136 (12.05%)	22 (11.11%)	114 (12.24%)	2.08	0.352
	7–10 days	255 (22.59%)	38 (19.19%)	217 (23.31%)		
	>10 days	738 (65.37%)	138 (69.7%)	600 (64.45%)		
Symmetrical		732 (64.84%)	126 (63.64%)	606 (65.2%)	0.152	0.743
Redness of joints		319 (28.26%)	52 (26.26%)	267 (28.68%)	0.470	0.493
Difficulty in daily activities		793 (70.24%)	150 (75.76%)	643 (69.1%)	3.98	0.061
Awaken from sleep due to pain		871 (65.7%)	153 (64%)	718 (66.1%)	0.361	0.548

Multivariate analysis of 20 major clinical variables between confirmed and probable cases showed no statistically significant differences between these two patient categories except for rash and swollen joint (S3 Table). This finding suggests that physicians were able to diagnose chikungunya cases effectively based only on typical clinical features during the outbreak.

### Health-economic impact

**Fig 2:**
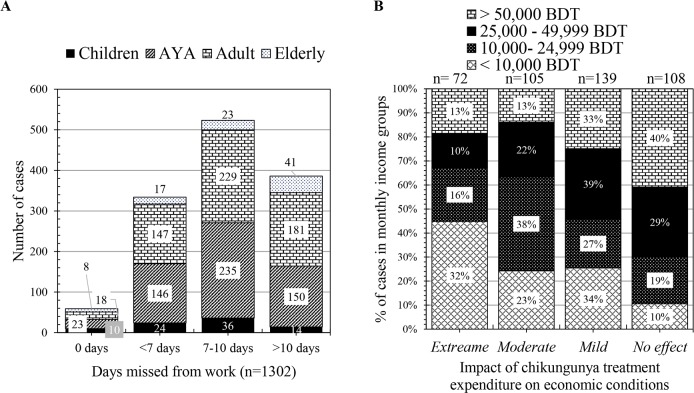
The economic impact of chikungunya infection on days misses from work (A) and rating versus income ranges as 100% staked column (B). Respondents were asked to rate the chikungunya healthcare expenditure on their economic conditions on a numeric rating scale of 1 to 10. Rating 8–10, 5–7, 2–4 and 1 is considered as extreme, moderate, mild and no impact on economic conditions of respondents, respectively. One column in B illustrates the relative percentage of cases from different income ranges. The exchange rate of 1 USD is about 82 BDT.

We estimated the overall treatment cost (including consultation fee, cost of laboratory tests, medicine, transport and special food) during the acute phase of chikungunya. We found that confirmed cases had to spend around BDT 8,192 (SD = 12,127.63) on an average compared to BDT 2,122 (SD = 3,422) for probable cases which are equivalent to $99.3(SD = 147) compared to $26 (SD = 41.5), respectively. Approximately 70% (n = 1,302) of the patients lost more than 7 productive days while 29.6% of them lost more than 10 days in the acute phase of the disease. Our analysis relying on 424 family heads suffering from chikungunya showed that economic impact was most prominent in low-income (< 10,000 BDT) categories (Fig 2, S4 Table).

### Quality of life

We assessed the quality of life (QoL) of 1,216 respondents who were 18 years and older. The Cronbach's alpha of WHOQOL-BREF was adequate (0.89) for all 26 questions. The average score was highest in the environmental health domain (mean = 11.43, SD = 2.52) followed by psychological domain (mean = 10.03, SD = 2.75), social relationship domain (mean = 10.02, SD = 2.94), and physical domain (mean = 8.32, SD = 2.33) (S5 Table). Pearson’s correlation coefficient showed a positive linear relationship between Q1 (represents overall QoL of WHOQOL-BREF) and all four domains separately (S6 Table). The strongest correlation was found between Q1 and the physical domain (r = 0.46), followed by the psychological domain (r = 0.36).

**Fig 3:**
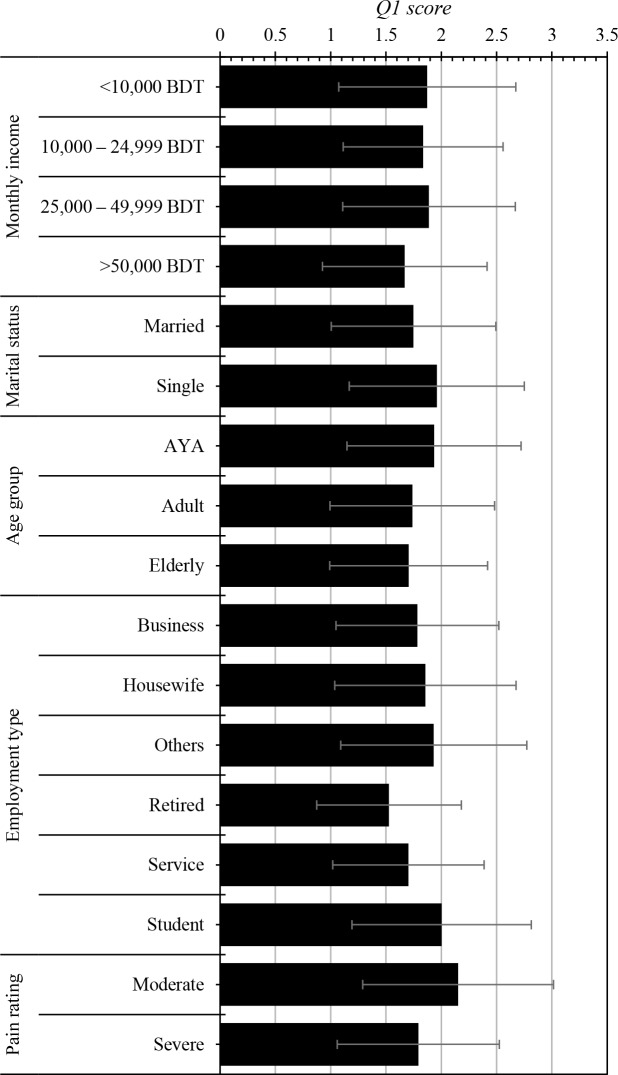
Impact of chikungunya infection on overall quality of life (Q1 of WHOQOL-BREF) among different socio-demographic status and pain rating.

Overall 83.2% patients responded ‘very low’ or ‘low’ on Q1 indicating a catastrophic impact on the quality of life during acute-phase CHIKV infection. Average Q1 scores were significantly affected by monthly income (p = 0.0032), marital status (p<0.0001), age (p<0.0001), employment type (p<0.0001). Patients with severe arthralgia had significantly lower QoL compared to mild to moderate arthralgia (p<0.0001) during the acute phase (Fig 3).

## Discussion

The first ever large-scale outbreak of chikungunya in Dhaka led to a rapid spread of infection across the city. To our knowledge, we have analyzed one of the largest samples of 1,326 cases to demonstrate the detail clinical profile of acute chikungunya infection. Our study also sheds light on the extent of economic impact on victim families and quality of life of the patients.

Both dengue and chikungunya viruses are transmitted by the same mosquito vector and often difficult to differentiate clinically. As discussed earlier, there was no warning of dengue outbreak by health authorities during our study period. Moreover, dengue incidences have been declining since the first large-scale dengue outbreak occurred in Dhaka in 2000 [40]. A seroprevalence study showed that around 80% individuals of Dhaka city had a past history of dengue infection [41]. Furthermore, a serology-based study revealed that approximately 6.1% (n = 271) of acute clinical cases (<7 days) of chikungunya was positive for dengue infection during recent chikungunya outbreak. Nearly 3.1% of these dengue positive patients were co-infected with CHIKV (obtained from personal communication; Professor Md. Akram Hossain, National Institute of Preventive & Social Medicine, Dhaka, Bangladesh).

On multivariate analysis, all clinical symptoms (except skin rash and swollen joints) were similar among confirmed and probable cases (Table 2, Table 3 and S3 Table). Compared to other studies conducted on self-reported [22,42–44] or hospitalized patients [45–47], a higher frequency of rash (69.6%) and swollen joints (52.1%) were documented in the present study.

In most reported outbreaks, sudden onset of fever was found to be the initial clinical symptom of chikungunya. However, joint (and/or muscle) pain preceded fever in more than 70% of patients in our study irrespective of age and sex (Table 2). In Kerala outbreak, joint pain was reported as the initial symptom in 17% patients, which varied among different age group reaching up to 77% in patients over 60 years [48]. This initial symptom of pain could be considered as the hallmark of chikungunya infection in Dhaka outbreak.

Severe arthropathy is the most consistent clinical feature of chikungunya infection. In our study, all patients experienced joint pain. Polyarthralgia was documented in about 56.3% of the patients while oligoarthralgia was present in 40.1% cases. In the present study, ankle (82.6%) and wrist (74.8%) were the most affected joints. In the acute phase, the frequency of incapacitating pain involving certain peripheral joints (ankle 82.6%, feet 63.8%, wrist 74.8%, fingers 73.2% and knee 74.5%) was found to be similar with that of French soldiers cohort and another study conducted in La Reunion [22,44] (S2 Table). In contrast, lower frequencies were reported in India and Suriname [42,45,48].

Over 85% of the patients (n = 1,326) experienced severe pain with a median NRS score of 8.3 throughout the acute phase. This finding is consistent with a study carried out in La Reunion [44]. About 70% of our patients faced problem in doing routine daily activities and about 65.7% reported sleep disturbance due to severe arthropathy (Table 3). The actual impact of chikungunya fever on daily life activities might be much higher than reported here because of poor health literacy in developing countries. Moreover, socio-culturally, patients often fail to explain their health conditions clearly; rather express them in terms of overall satisfaction from a spiritual standpoint. What it means is that even though they are clinically suffering, people would report that they are alright. The overall severity and the extent of arthralgia related manifestations suggest an aggressive strain of chikungunya virus probably circulated in Dhaka. The sequencing of the viral strain is warranted to find out the lineage of chikungunya virus.

The severity of certain clinical manifestations of chikungunya might depend on several factors including age, gender, immune status, genetic predisposition and co-morbid conditions [49]. Bivariate analysis showed that children (<15 years) tended to have a higher proportion of oligo-arthralgia and skin rash; while morning stiffness, severity, and duration of pain were proportionally lower among children as compared to other age groups. Joint swelling was most commonly noted in elderly patients (60+ years), while the severity of pain was highest among adults (30–59 years). In our study, the number of male participants was higher than female participants. Female patients experienced a higher incidence of skin rash, itching, joint-swelling compared to their male counterparts (S1 Fig).

We found that chikungunya infection caused significant loss of productivity due to absenteeism from job, household work and school. Over 95% of the respondents (including confirmed and probable cases, n = 1,302) were mostly confined to sickbed. As a consequence, 29.6% of the patients lost more than 10 days of productivity during the acute phase (Fig 2). Notably, no national health insurance system exists in Bangladesh and therefore, all treatment costs were considered as out-of-pocket expenditures of the patients. Considering socio-economic conditions, a significant amount of money had to be spent for treatment purposes. We have not estimated the overall economic burden of the loss of productivity due to this chikungunya outbreak. However, we have attempted to paint a picture of the economic impact on victim families based on responses to a rating scale. Our analysis suggests that low income (<$303 per month) families are more likely to face significant economic pressure. Particularly, families of daily workers were the worst hit while there was no significant impact on the families in the higher income category (>$606 per month). Anecdotal evidence suggests that many daily or menial workers lost their jobs due to long absenteeism. It is indispensable to estimate the overall disease burden through systematic epidemic studies to determine the real economic burden of an outbreak like this.

Majority of the patients in our study reported low to very low overall quality of life. Although males had slightly higher average scores than females, the difference was not statistically significant (p = 0.138). QoL was significantly lower in patients with severe pain compared to those with moderate pain. Elderly patients reported lower average QoL scores compared to <60 years. In particular, patients in the highest income bracket (BDT 50,000 per month; >$606 per month) reported the lowest average overall score (1.66, p = 0.003). This could be due to the fact that most of the respondents in the higher income group are older. Overall QoL scores differed significantly between different job categories (Fig 3). Not surprisingly, students reported the highest average score (2.0) which is consistent with our findings that younger patients who are mostly students, reported higher scores compared to their older counterparts. The jobless and dependents showed an overall score of 1.93. Interestingly, housewives reported higher QoL score (1.86) compared to those of businessmen (1.78) and service holders (1.70). It would be of interest to explore the interplay between gender and various socio-cultural factors in terms of quality of life.

Our study has some limitations. First, the participants were purposively selected using social connections. Even though it is not uncommon to adopt a non-random sampling design in such circumstances (considering Bangladesh being one of the healthcare-resource-limited countries), the results should be interpreted after taking this into consideration. Second, the representation of confirmed cases was relatively low (18%). This could be explained by the limited availability of diagnostic facility along with high cost of the tests during CHIKV outbreak. Third, because of the retrospective data collection scheme, there could be recall bias. However, since our study was conducted during the very end of the peak of outbreak, it is likely that the recall bias was minimum. Lastly, it is possible that some participants may have overvalued some clinical symptoms due to massive media coverage of the outbreak.

Our work represents one of the largest samples (n = 1,326) studied so far around the world describing the clinical profile of chikungunya infection. This study demonstrates the severity of clinical symptoms during the acute phase and how it has impacted on productivity and quality of life of the affected individuals. We found joint pain prior to fever as a unique symptom in the Dhaka (2017) outbreak. A possible reason could be a novel viral strain that warrants future molecular investigations. Our findings support that during an established outbreak, CHIKV patients can effectively be identified using a set of easily recognisable clinical criteria (i.e. syndromic approach) without lab confirmation; an approach also suggested by others [50,43]for resource-constrained developing countries. We believe our study would play a pivotal role in devising an effective syndromic surveillance system for CHIKV in Bangladesh that would allow early detection of future outbreaks and timely public health interventions.

## Supporting information

S1 Checklist STROBE checklist. (DOC)

S1 Fig Variability of different clinical conditions among different sexes (A) and age groups (B-E). Respondents aged <15 years, 15–29 years, 30–59 years and >59 years denotes as Children, AYA, Adult and Elderly patients, respectively. A two-tailed p-value smaller than 0.05 was considered statistically significant.
(PDF)

S1 Table Additional clinical profile of chikungunya patients (n = 1326) in Bangladesh. (DOCX)

S2 Table Additional arthralgia profile of chikungunya patients (n = 1129, cases with past history of arthritis were excluded) in Bangladesh. (DOCX)

S3 Table Multivariate analysis of major clinical variables. (DOCX)

S4 Table Impact of chikungunya health expenditure on economic conditions of family heads (n = 424) according to different socio-demographic status. (DOCX)

S5 Table Quality of life: Calculated domain scores. (DOCX)

S6 Table Pearson’s correlation coefficients between Q1 and the four domains. (DOCX)
