# Supplementary figures and images for: Chikungunya outbreak (2017) in Bangladesh: Clinical profile, economic impact and quality of life during the acute phase of the disease
Source: PLoS Negl Trop Dis. 2018 Jun 6;12(6):e0006561. doi: 10.1371/journal.pntd.0006561 (PMC6025877; doi:10.1371/journal.pntd.0006561)

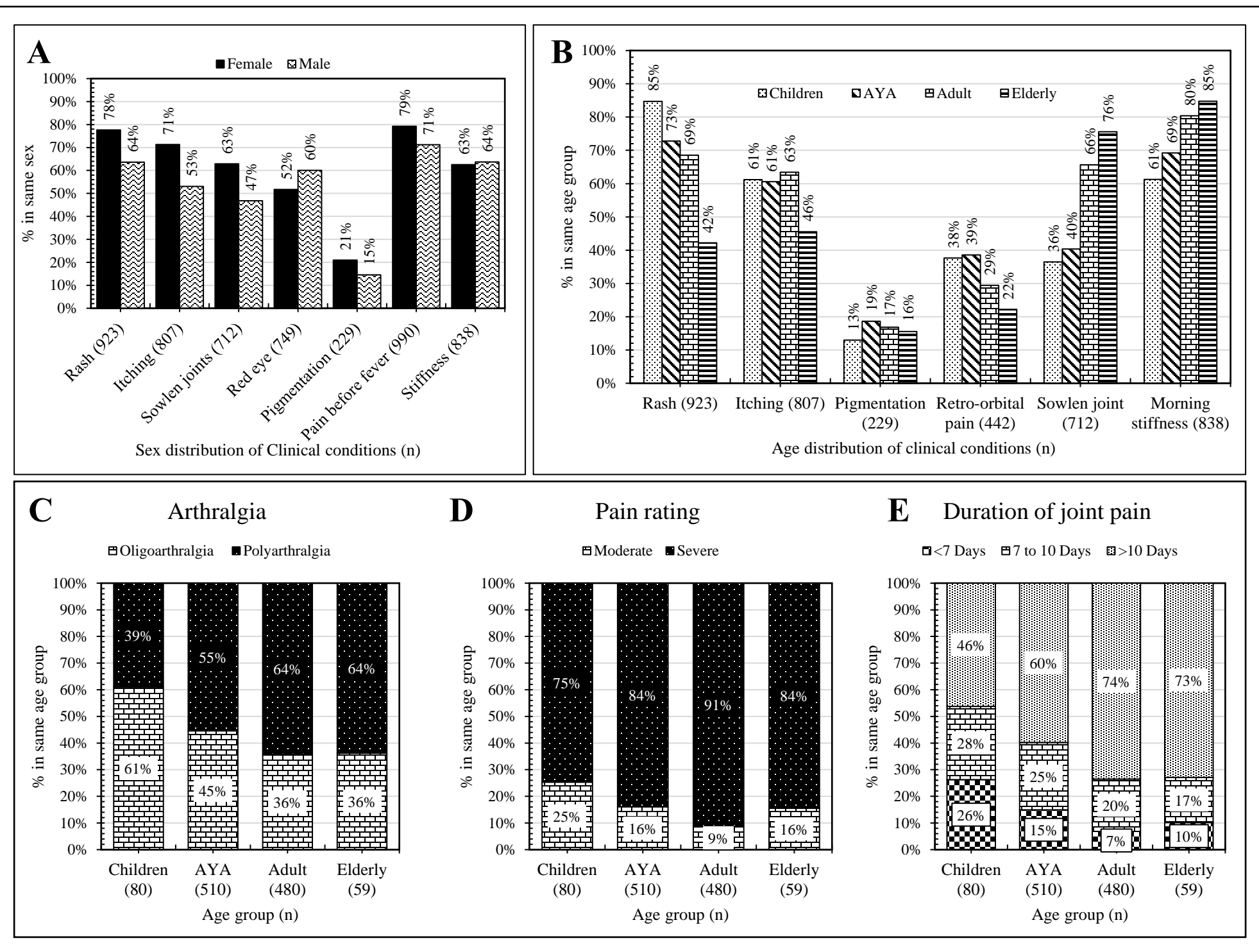

**S1 Fig.**

Supplement: S1 Fig — Variability of different clinical conditions among different sexes (A) and age groups (B-E). Respondents aged <15 years, 15–29 years, 30–59 years and >59 years denotes as Children, AYA, Adult and Elderly patients, respectively. A two-tailed p-value smaller than 0.05 was considered statistically significant. (PDF) [file pntd.0006561.s002.pdf]
