# Supplementary material for: Chikungunya outbreak (2017) in Bangladesh: Clinical profile, economic impact and quality of life during the acute phase of the disease
Source: PLoS Negl Trop Dis. 2018 Jun 6;12(6):e0006561. doi: 10.1371/journal.pntd.0006561 (PMC6025877; doi:10.1371/journal.pntd.0006561)
Supplement: S1 Table — (DOCX) [file pntd.0006561.s003.docx]

**S1 Table.** Additional clinical profile of chikungunya patients (n=1326) in Bangladesh.

| Variable | | | Total cases | | Confirmed cases | Probable cases | | P |
| --- | --- | --- | --- | --- | --- | --- | --- | --- |
| Fever | | | | | | | | |
|  | | Fever | 1326 (100%) | 239 (100%) | | 1087 (100%) |  | |
|  | | Maximum temp (Mean) | 103.66 | 103.64 | | 103.66 | 0.638 | |
|  | | Mean Duration (days) | 4.88 | 4.50 | | 4.96 | 0.336 | |
|  | | Chills | 1120 (84.5%) | 197 (82.42%) | | 923 (84.91%) | 0.337 | |
|  | | Sudden fever | 904 (68.17%) | 163 (68.2%) | | 741 (68.16%) | 0.992 | |
| Cough or runny nose | | | 302 (22.77%) | 35 (14.64%) | | 267 (24.56%) | 0.001 | |
| Headache was prominent than joint pain | | | 266 (20.1%) | 39 (16.3%) | | 227 (20.9%) | 0.111 | |
| Eye problems | | | | | | | | |
|  | Red eye | | 749 (56.5%) | 127 (53.1%) | | 622 (57.2%) | 0.249 | |
|  | Retro-orbital pain | | 442 (33.3%) | 66 (27.6%) | | 376 (34.6%) | 0.038 | |
| Gastrointestinal problems | | | | | | | | |
|  | Loss of appetite | | 926 (69.8) | 168 (70.3%) | | 758 (69.7%) | 0.864 | |
|  | Nausea | | 795 (60%) | 150 (62.8%) | | 645 (59.3%) | 0.328 | |
|  | Diarrhea | | 333 (25.1%) | 66 (27.6%) | | 267 (24.6%) | 0.325 | |
|  | Abdominal cramp | | 174 (13.1%) | 33 (13.8%) | | 141 (13%) | 0.729 | |
|  | Irregular bowel movement | | 136 (10.3%) | 20 (8.4%) | | 116 (10.7%) | 0.288 | |
|  | Vomiting | | 141 (10.6%) | 35 (14.6%) | | 106 (9.8%) | 0.026 | |
|  | Others | | 10 (0.8%) | 0 (0%) | | 10 (0.9%) | 0.137 | |
| Other symptoms | | | | | | | | |
|  | Chest pain | | 170 (12.8%) | 29 (12.1%) | | 141 (13%) | 0.726 | |
|  | Blurred vision | | 230 (17.3%) | 36 (15.1%) | | 194 (17.8%) | 0.303 | |
|  | Memory loss | | 103 (7.8%) | 22 (9.2%) | | 81 (7.5%) | 0.359 | |
|  | Others | | 74 (5.6%) | 20 (8.4%) | | 54 (5%) | 0.038 | |
| Oral ulcer | | | 414 (31.2%) | 76 (31.8%) | | 338 (31.1%) | 0.832 | |
| Bleeding | | | 40 (3%) | 4 (1.7%) | | 36 (3.3%) | 0.18 | |
