# Supplementary material for: Chikungunya outbreak (2017) in Bangladesh: Clinical profile, economic impact and quality of life during the acute phase of the disease
Source: PLoS Negl Trop Dis. 2018 Jun 6;12(6):e0006561. doi: 10.1371/journal.pntd.0006561 (PMC6025877; doi:10.1371/journal.pntd.0006561)
Supplement: S2 Table — (DOCX) [file pntd.0006561.s004.docx]

**S2 Table****.** Additional arthralgia profile of chikungunya patients (n=1129, cases with past history of arthritis were excluded) in Bangladesh

| Variable | | Total cases | Confirmed cases | Probable cases | χ^2^ | P |
| --- | --- | --- | --- | --- | --- | --- |
| Major joint affected | | | | | | |
|  | Finger | 826 (73.2%) | 135 (68.2%) | 691 (74.2%) | 3.033 | 0.082 |
|  | Wrist | 845 (74.8%) | 144 (72.7%) | 701 (75.3%) | 0.572 | 0.449 |
|  | Spine | 589 (52.2%) | 91 (46.0%) | 498 (53.5%) | 3.711 | 0.054 |
|  | Knee | 841 (74.5%) | 147 (74.2%) | 694 (74.5%) | 0.008 | 0.93 |
|  | Ankle | 932 (82.6%) | 165 (83.3%) | 767 (82.4%) | 0.102 | 0.749 |
|  | Feet | 720 (63.8%) | 114 (57.6%) | 606 (65.1%) | 3.99 | 0.046 |
|  | Shoulder | 412 (36.5%) | 73 (36.9%) | 339 (36.4%) | 0.015 | 0.904 |
|  | Chest | 208 (18.4%) | 37 (18.7%) | 171 (18.4%) | 0.011 | 0.916 |
|  | Others | 59 (5.2%) | 11 (5.6%) | 48 (5.2%) | 0.053 | 0.818 |
| Swelling of joints | | | | | | |
|  | Finger | 291 (25.8%) | 53 (26.8%) | 238 (25.6%) | 0.124 | 0.725 |
|  | Wrist | 221 (19.6%) | 41 (20.7%) | 180 (19.3%) | 0.196 | 0.658 |
|  | Ankle | 416 (36.8%) | 91 (46%) | 325 (34.9%) | 8.569 | 0.003 |
|  | Feet | 256 (22.7%) | 47 (23.7%) | 209 (22.4%) | 0.155 | 0.694 |
