# Supplementary material for: Chikungunya outbreak (2017) in Bangladesh: Clinical profile, economic impact and quality of life during the acute phase of the disease
Source: PLoS Negl Trop Dis. 2018 Jun 6;12(6):e0006561. doi: 10.1371/journal.pntd.0006561 (PMC6025877; doi:10.1371/journal.pntd.0006561)
Supplement: S3 Table — (DOCX) [file pntd.0006561.s005.docx]

# S3 Table. Multivariate analysis of major clinical variables.

Crude and Adjusted Odds Ratios with 95% confidence limits. These are all binary variables with ‘No’ as the reference category.

|  | Crude Odds Ratio | | | Adjusted Odds Ratio | | |
| --- | --- | --- | --- | --- | --- | --- |
|  | OR | Lower 95% CL | Upper 95% CL | Adj OR | Lower 95% CL | Upper 95% CL |
| (Intercept) |  |  |  | 0.20 | 0.09 | 0.47 |
| Chills | 0.83 | 0.58 | 1.22 | 0.85 | 0.54 | 1.36 |
| Pain before fever | 0.96 | 0.70 | 1.33 | 0.82 | 0.56 | 1.21 |
| Rash | 1.72 | 1.23 | 2.41 | 1.90 | 1.24 | 2.98 |
| Itching | 1.06 | 0.79 | 1.41 | 1.08 | 0.74 | 1.58 |
| Headache | 0.67 | 0.47 | 0.97 | 0.71 | 0.48 | 1.06 |
| Retro-orbital pain | 0.71 | 0.49 | 1.02 | 0.79 | 0.52 | 1.18 |
| Red eye | 0.90 | 0.65 | 1.24 | 0.94 | 0.65 | 1.34 |
| Loss of appetite | 1.04 | 0.73 | 1.48 | 1.12 | 0.78 | 1.62 |
| Nausea | 1.19 | 0.86 | 1.67 | 1.24 | 0.87 | 1.77 |
| Diarrhea | 1.15 | 0.79 | 1.65 | 1.18 | 0.80 | 1.73 |
| Bleeding | 0.31 | 0.05 | 1.05 | 0.31 | 0.05 | 1.06 |
| Myalgia | 0.86 | 0.61 | 1.21 | 0.93 | 0.65 | 1.34 |
| Spine | 0.82 | 0.62 | 1.10 | 1.06 | 0.74 | 1.50 |
| Swelling of joints | 1.72 | 1.28 | 2.33 | 1.73 | 1.20 | 2.51 |
| Joint stiffness | 1.00 | 0.73 | 1.42 | 0.91 | 0.61 | 1.36 |
| Symmetrical pain | 1.02 | 0.76 | 1.38 | 1.02 | 0.72 | 1.47 |
| Oligoarthralgia | 1.26 | 0.92 | 1.73 | 1.09 | 0.73 | 1.63 |
| Polyarthralgia | 0.69 | 0.49 | 0.99 | 0.71 | 0.46 | 1.12 |
| Edema | 1.60 | 1.05 | 2.40 | 1.39 | 0.88 | 2.16 |
| Redness of joints | 0.97 | 0.70 | 1.33 | 0.73 | 0.48 | 1.08 |

# 
