# Supplementary material for: Chikungunya outbreak (2017) in Bangladesh: Clinical profile, economic impact and quality of life during the acute phase of the disease
Source: PLoS Negl Trop Dis. 2018 Jun 6;12(6):e0006561. doi: 10.1371/journal.pntd.0006561 (PMC6025877; doi:10.1371/journal.pntd.0006561)
Supplement: S4 Table — (DOCX) [file pntd.0006561.s006.docx]

# S4 Table. Impact of chikungunya health expenditure on economic conditions of family heads (n=424) according to different socio-demographic status.

| Variable | | Extreme (rating 8-10) | Moderate (rating 5-7) | Mild (rating 2-4) | None (rating 1) | Total | χ ^2^ | | p |
| --- | --- | --- | --- | --- | --- | --- | --- | --- | --- |
|  |  | 72 | 105 | 139 | 108 | 424 | |  |  |
| Case type | | | | | | | | | |
|  | Probable cases | 58 (80.6%) | 85 (81%) | 105 (76%) | 86 (80%) | 334 (79%) | 1.35 | | 0.72 |
|  | Confirmed cases | 14 (19.4%) | 20 (19%) | 34 (24%) | 22 (20%) | 90 (21%) |  | |  |
| Age group | | | | | | | | | |
|  | AYA (15-29) | 15 (20.8%) | 10 (10%) | 13 (9%) | 13 (12%) | 51 (12%) | 16.43 | | 0.06 |
|  | Adult (30-59) | 52 (72.3%) | 79 (75%) | 108 (78%) | 70 (64%) | 309 (73%) |  | |  |
|  | AYA (15-29) | 5 (6.9%) | 16 (15%) | 18 (13%) | 25 (23%) | 64 (15%) |  | |  |
| Gender | | | | | | | | | |
|  | Female | 13 (18.1%) | 11 (10%) | 19 (14%) | 19 (18%) | 62 (15%) | 2.99 | | 0.39 |
|  | Male | 59 (81.9%) | 94 (90%) | 120 (86%) | 89 (82%) | 362 (85%) |  | |  |
| Occupation | | | | | | | | | |
|  | Business | 15 (20.8%) | 22 (21%) | 35 (25%) | 23 (21%) | 95 (22%) | 23.70 | | 0.02 |
|  | Housewife | 3 (4.2%) | 2 (2%) | 9 (6%) | 9 (8%) | 23 (5%) |  | |  |
|  | Retired | 2 (2.8%) | 7 (7%) | 6 (4%) | 13 (12%) | 28 (7%) |  | |  |
|  | Service | 51 (70.8%) | 67 (64%) | 78 (56%) | 62 (57%) | 258 (61%) |  | |  |
|  | Others | 1 (1.4%) | 7 (7%) | 11 (8%) | 1 (1%) | 20 (5%) |  | |  |
| Housing (apartment) type | | | | | | | | | |
|  | Managed | 41 (56.9%) | 60 (57%) | 76 (55%) | 84 (78%) | 261(62%) | 16.52 | | 0.01 |
|  | Unman-aged | 29 (40.3%) | 43 (41%) | 60 (43%) | 23 (21%) | 155(37%) |  | |  |
|  | Other | 2 (2.8%) | 2 (2%) | 3 (2%) | 1 (1%) | 8 (2%) |  | |  |
| Highest education completed | | | | | | | | | |
|  | Graduate | 32 (44.4%) | 45 (43%) | 69 (50%) | 70 (65%) | 216 (51%) | 14.25 | | 0.11 |
|  | Illiterate | 7 (9.7%) | 8 (8%) | 11 (8%) | 4 (4%) | 30 (7%) |  | | |
|  | Primary | 16 (22.2%) | 23 (22%) | 29 (21%) | 13 (12%) | 81 (19%) |  |  |  |
|  | Secondary | 17 (23.6%) | 29 (28%) | 30 (22%) | 21 (19%) | 97 (23%) |  |  |  |
| Monthly income (BDT) | | | | | | | | | |
|  | <10,000 | 25 (34.7%) | 18 (17%) | 26 (19%) | 8 (7%) | 77 (18%) | 50.83 | | 0.00 |
|  | 10,000-24,999 | 21 (29.2%) | 49 (47%) | 35 (25%) | 25 (23%) | 130 (31%) |  | | |
|  | 25,000-49,999 | 11 (15.3%) | 23 (22%) | 41 (29%) | 30 (28%) | 105 (25%) |  |  |  |
|  | >=50,000 | 15 (20.8%) | 15 (14%) | 37 (27%) | 45 (42%) | 112 (26%) |  |  |  |

# 
