# Supplementary material for: Chikungunya outbreak (2017) in Bangladesh: Clinical profile, economic impact and quality of life during the acute phase of the disease
Source: PLoS Negl Trop Dis. 2018 Jun 6;12(6):e0006561. doi: 10.1371/journal.pntd.0006561 (PMC6025877; doi:10.1371/journal.pntd.0006561)
Supplement: S5 Table — (DOCX) [file pntd.0006561.s007.docx]

# S5 Table. Quality of life: calculated domain scores.

|  | Mean | SD | Median | Min | Max | IQR | Cronbach's alpha coefficient |
| --- | --- | --- | --- | --- | --- | --- | --- |
| Domain 1 (Physical) | 8.32 | 2.33 | 8 | 4 | 17.14 | 2.86 | 0.50 |
| Domain 2 (Psychological) | 10.03 | 2.75 | 10 | 4 | 20 | 4 | 0.49 |
| Domain 3 (Social Relationship) | 10.02 | 2.94 | 13.33 | 4 | 20 | 4 | 0.61 |
| Domain 4 (Environmental Health) | 11.43 | 2.52 | 11.5 | 4.5 | 19 | 3 | 0.64 |

# 
