# Supplementary material for: Chikungunya outbreak (2017) in Bangladesh: Clinical profile, economic impact and quality of life during the acute phase of the disease
Source: PLoS Negl Trop Dis. 2018 Jun 6;12(6):e0006561. doi: 10.1371/journal.pntd.0006561 (PMC6025877; doi:10.1371/journal.pntd.0006561)
Supplement: S6 Table — (DOCX) [file pntd.0006561.s008.docx]

**S6 Table**. Pearson’s Correlation Coefficients between Q1 and the four domains.

|  | Q1 (Overall QoL) | Domain 1 | Domain 2 | Domain 3 | Domain 4 |
| --- | --- | --- | --- | --- | --- |
| Domain 1 (Physical) | 0.46 | - | - | - | - |
| Domain 2 (Psychological) | 0.36 | 0.61 | - | - | - |
| Domain 3 (Social Relationship) | 0.17 | 0.25 | 0.26 | - | - |
| Domain 4 (Environmental Health) | 0.12 | 0.21 | 0.21 | 0.27 | - |

# 
